# The Regular Interaction Pattern among Odorants of the Same Type and Its Application in Odor Intensity Assessment

**DOI:** 10.3390/s17071624

**Published:** 2017-07-13

**Authors:** Luchun Yan, Jiemin Liu, Shen Jiang, Chuandong Wu, Kewei Gao

**Affiliations:** 1 School of Materials Science and Engineering, University of Science and Technology Beijing, Beijing 100083, China; yanluchun@126.com (L.Y.); kwgao@mater.ustb.edu.cn (K.G.); 2 School of Chemistry and Biological Engineering, University of Science and Technology Beijing, Beijing 100083, China; janggemini@163.com (S.J.); dong1035@126.com (C.W.)

**Keywords:** aldehydes, e-nose, esters, odor intensity, vector model

## Abstract

The olfactory evaluation function (e.g., odor intensity rating) of e-nose is always one of the most challenging issues in researches about odor pollution monitoring. But odor is normally produced by a set of stimuli, and odor interactions among constituents significantly influenced their mixture’s odor intensity. This study investigated the odor interaction principle in odor mixtures of aldehydes and esters, respectively. Then, a modified vector model (MVM) was proposed and it successfully demonstrated the similarity of the odor interaction pattern among odorants of the same type. Based on the regular interaction pattern, unlike a determined empirical model only fit for a specific odor mixture in conventional approaches, the MVM distinctly simplified the odor intensity prediction of odor mixtures. Furthermore, the MVM also provided a way of directly converting constituents’ chemical concentrations to their mixture’s odor intensity. By combining the MVM with usual data-processing algorithm of e-nose, a new e-nose system was established for an odor intensity rating. Compared with instrumental analysis and human assessor, it exhibited accuracy well in both quantitative analysis (Pearson correlation coefficient was 0.999 for individual aldehydes (*n* = 12), 0.996 for their binary mixtures (*n* = 36) and 0.990 for their ternary mixtures (*n* = 60)) and odor intensity assessment (Pearson correlation coefficient was 0.980 for individual aldehydes (*n* = 15), 0.973 for their binary mixtures (*n* = 24), and 0.888 for their ternary mixtures (*n* = 25)). Thus, the observed regular interaction pattern is considered an important foundation for accelerating extensive application of olfactory evaluation in odor pollution monitoring.

## 1. Introduction

In recent years, air quality has been listed as an important objective in building an environment-friendly and livable city. Odor pollution especially causes wide public concerns because it will seriously lower the quality of life [1,2]. These typical pollution sources including painting plants, petrochemical factories, and building materials factories in industrial parks normally make up a major proportion of the total odor pollutants emission, and they also occupy an irreplaceable role in promoting the city’s economy development [3]. Against this background, specific air monitoring systems and waste gas management methods have been abundantly developed to balance it out [4,5,6].

As one of the research focuses in air quality monitoring and assessment, the electronic nose (e-nose) has been successfully applied in many related fields [7,8,9,10,11,12]. Both sensor signal process and quantitative analysis abilities of e-nose are apparently improved, especially with the rapid development of artificial neural networks (ANNs) in recent years [13,14]. However, e-nose still can’t perform an effective odor intensity (OI) rating which is an important criteria in the assessment of odor pollution levels. But similar olfactory assessment like perfume diagnose has been successfully performed on the basis of signals from the sensor array of an electronic nose [15]. It is mainly attributed to the significantly different odor-causing ability of volatile organic compounds (VOCs) [16]. For example, sulfur organic compounds could easily cause strong sensory stimulation at very low concentrations while substances like ethanol could hardly be detected at the same concentration [17]. Besides, the phenomenon of odor interaction is generally observed in odor mixtures, and it is influenced by many factors [18,19]. For instance, the OI of a binary mixture was lower than the summation of its unmixed constituents’ odor intensities (i.e., odor counteraction) [20]. However, comparing with restrictions on traditional olfactory tests (e.g., experienced assessors and well ventilated testing environments), e-nose still has distinct advantages including on-line monitoring, good stability, and high adaptability. Thus empirical models, which aims at establishing a conversion method between the OI of an odor mixture with its constituents’ chemical concentrations, are supposed to be helpful to enhance the e-nose capacity [21,22]. In a previous study, the feasibility of OI rating with e-nose had been successfully demonstrated by embedding empirical models as a part of its signal processing procedure [23]. Besides through comparison among several different empirical models, an extended vector model showed a pleasing degree of matching of OI rating results with human assessors.

The vector model is a well acknowledged model dealing with the odor interaction in odor mixtures, and it suggests that the perceived intensity of mixtures equals the vector sum of perceived intensities of their unmixed components [24]. As the OI of each unmixed component still needs normal olfactory assessment, a novel modification method of the vector model was proposed in our previous study [25]. The modified vector model (MVM) associated the OI of a mixture with its constituents’ logarithm values of odor activity value (OAV) which could be directly calculated based on their chemical concentrations. On the other hand, researches of empirical models are mostly confined to individual stimuli or specific mixtures in the literatures [26]. It will seriously limit the combined application of themselves with other techniques like e-nose. However, in investigation of the MVM, it was found that a group of mixtures consisted of different aromatic hydrocarbons all basically complied with the same model. Thus these substances exhibited a similar odor interaction pattern in their mixtures, and it was supposed to be determined by their alike molecular features (e.g., same functional group, similar chemical structure, and analogical odor type) [25,27]. However, whether the regular odor interaction pattern also applies to other groups of odorants has not been verified. If the MVM is confirmed for broad application, it will apparently extend their combined applications in related fields.

This study aims at proposing a more generic method of odor intensity prediction especially for odor mixtures. At first, practicability of the MVM to aldehydes and esters were individually tested. Then, the phenomenon of a regular interaction pattern among odorants of the same type was verified. Since odor intensity of an odor mixture could be directly converted to its constituents’ chemical concentrations in use of the MVM, it was planned to combine it with e-nose for olfactory evaluation. After establishing an e-nose system which consisted of a gas sensor array and data processing algorithms, the MVM was also embedded in it as a part of the data processing algorithm for odor intensity assessment. Performance of the established e-nose system would be verified by comparing with both gas chromatography and human assessors. Based on the above research, it indicates the applicable feasibility of portable devices (e.g., e-nose) and on-line monitoring systems in odor intensity assessment. With the development of olfactory evaluation function in these devices, the automatic level of monitoring and supervision of environmental pollution will be significantly improved.

## 2. Materials and Methods

### 2.1. Stimuli and Assessors

All the reagents (Table 1) used in the experiments were purchased from J & K Scientific (Beijing, China). Standard gas of each odorant was firstly prepared in an odor-free bag (3 L volume and full of odor-free air) with an evaporation method. Then the odor sample was prepared through transferring a certain amount of standard gas to a new plastic bag (made of odorless polyester film, Sinodour, Tianjin, China) and diluted with purified air.

A sensory panel (eight assessors, four males and four females) were recruited from the University of Science and Technology Beijing. Their ages ranged from 21 to 27 years (mean = 24 years), and all of them had participated in several experiments using the same procedures and apparatus. In order to identify the applicability of MVM under different rating standards, two common odor intensity referencing scales (Table 2, twelve levels for aldehydes, and eight levels for esters) were used in this study [32]. Before the test, standard water solutions of *n*-butanol were prepared according to the Odor intensity referencing scales (OIRS). Then, assessors sniffed the odor sample and then they were asked to point out the best match on the OIRS which exhibited an odor intensity matching that of the sample. The mean value of assessors’ rating results to an odor sample was calculated as its measured odor intensity. Tests took place in an odor-free and well ventilated room.

### 2.2. The Modified Vector Model

According to the vector model, OI of an odor mixture is usually related with the odor intensities of its unmixed constituents as the following equations:

(1) $${OΙ}_{\mathrm{ab}}^{2}={OΙ}_{a}^{2}+{OΙ}_{b}^{2}+2\times \cos\alpha_{\mathrm{ab}}\times {OΙ}_{a}\cdot {OΙ}_{b}$$

(2) $$\begin{array}{ll} {OΙ}_{\mathrm{abc}}^{2}={OΙ}_{a}^{2}+{OΙ}_{b}^{2}+{OΙ}_{c}^{2} & +2\times \cos\alpha_{\mathrm{ab}}\times {OΙ}_{a}\cdot {OΙ}_{b}\\ & +\;2\times \cos\alpha_{\mathrm{ac}}\times {OΙ}_{a}\cdot {OΙ}_{c}\\ & +\;2\times \cos\alpha_{\mathrm{bc}}\times {OΙ}_{b}\cdot {OΙ}_{c} \end{array}$$

where the interaction coefficient cosα represents the degree of interaction between these two unmixed constituents. In the previous research of aromatic hydrocarbons, the modifications of vector models were mainly proposed based on two observed phenomena [25]. The first one was a linear relation between OI of individual odorants and its corresponding lnOAV values:

(3) OI=k·lnOAV+b

where OAV is calculated as a ratio between a stimulus’s chemical concentration and its odor threshold (i.e., OAV = C/C_Thr._), and *k* and *b* are constants. The odor thresholds of employed odorants in this study were firstly measured according to the standard test method [32]. As shown in Table 1, the measured odor thresholds and another two sets of reported odor thresholds are listed. Since the composition of a sensory panel (e.g., quantity, age, and gender) usually influences the odor threshold measurement, differences between the two sets of reported odor thresholds are generally accepted. Through comparison, the measured odor thresholds were considered at reasonable intervals. Thus, the measured odor thresholds would be employed in the following calculations. On the basis of the above results, OI of any unmixed constituent in the vector model formulas would be directly calculated on the basis of its chemical concentration value which could be measured by instrumental analysis. The second phenomenon was that any binary mixture of aromatic hydrocarbons all basically followed the same linear relation between OI of the mixture (OI_mix._) and summation of its unmixed constituents’ odor intensities (OI_sum._). Normally, the value of cosα is measured and calculated as depicted in Equation (4) when two constituents of equal perceived intensities were mixed.

(4) $$m=\cos\alpha_{\mathrm{ab}}=\frac{{OΙ}_{\mathrm{ab}}^{2}-{OΙ}_{a}^{2}-{OΙ}_{b}^{2}}{2{OΙ}_{a}\cdot {OΙ}_{b}}$$

Then through the use of the above linear relation, the OI of odor mixture (i.e., OI_ab_) in Equation (4) could be displaced with its constituents’ odor intensities (i.e., OI_a_ and OI_b_). As a result, the cosα (e.g., cosα_ab_, cosα_ac_, cosα_bc_ in Equation (2)) was proved to be a fixed value (e.g., *m*) for those binary mixtures which consisted of the same kind of stimuli.

### 2.3. Experimental Apparatus

The MVM research used a similar e-nose system as descripted in our previous study [23]. It mainly consisted of a sensory array and a customized single chip microcontroller (SCM). Sensors were usually supposed to have different degrees of selectivity and sensitivity to target substances [33]. Thus, sensors were selected through a series of evaluation tests (i.e., response value, response time, recovery time, and linear response). Sensor response was transferred to digital signals and they were further processed by a back-propagation (BP) neural network algorithm (one of the most important ANNs algorithms) which had been embedded in the SCM. Since the BP algorithm assumed quantitative analysis (outputting chemical concentration of each constituent), the MVM (converting constituents’ chemical concentrations to the OI of the corresponding odor mixture) also could be embedded in the algorithm to cooperate with it for OI rating.

In the aspect of BP algorithm, an initial BP neural network was built with default parameters. It consisted of an input layer (neuron quantity equals to the number of employed gas sensors), hidden layers (number of layers and quantity of neurons in each layer would be determined through training), and an output layer (each neuron corresponded to a specific substance). Based on a training database, the BP algorithm would be trained to obtain optimized parameters. During the training, its algorithm parameters (i.e., weight values and threshold values) would be automatically adjusted to acquire a minimum error between the predicted output and the target output.

### 2.4. Experimental Procedures

This study mainly consisted of three parts. In the first part, a set of odor samples at different concentration levels and various mixing ratios were prepared (*n* = 6 for each single odorant of A, P, B, EA, BA, and EB; *n* = 5 for each binary mixture of A + P, A + B, P + B, EA + BA, BA + EB, and EA + EB). Chemical concentration and odor intensity of each sample were individually measured. For samples of a single odorant, the lnOAV value was calculated on the basis of its chemical concentration and measured odor threshold (Table 1). Then, their linear relation between OI and lnOAV was verified. For a sample of a binary mixture, OI of the mixture and its unmixed constituents’ odor intensities were all measured by the sensory panel. After that, the linear relationship of OI_mix._ − OI_sum._ was also explored. Based on the above two linear relations, the MVM of aldehydes and esters were investigated respectively. In the second part, a group of gas sensors were individually tested when they were exposed to odor samples of single A, P, and B, respectively. According to the concentration dependence of sensor response, sensors were selected to establish a sensory array. Then, another set of odor samples (*n* = 8 for A, P, B; *n* = 25 for each binary mixture; *n* = 50 for their ternary mixture) were measured by both the gas chromatography and the e-nose as depicted in Section 2.3. Results like chemical concentration and sensor signals were collected as a training database. Based on it, the parameters of the BP algorithm were optimized through a training and then an optimized e-nose system was established. Finally in the third part, the MVM of aldehydes was embedded in to the e-nose as part of its data processing algorithm. Both quantitative analysis and OI rating performances of the e-nose system were verified through tests to a group of new prepared odor samples.

For each odor sample, its odor intensity was calculated as the average of all the scores rated by assessors in the sensory panel. The chemical concentrations of odor samples were measured by gas chromatography (GC-2014, Shimadzu, Kyoto, Japan) with the flame ionization detector (FID) and an Rtx-5 capillary column (30 m × 0.25 mm ID, 0.5 μm film thickness). The carrier gas was nitrogen (≥99.999%) at 1.0 mL/min and the injection port was 200 °C. The column oven temperature was set to 50 °C for 3 min and up to 200 °C at 10 °C·min^−1^ and held for 5 min.

## 3. Results and Discussion

### 3.1. The MVM of Aldehydes

**Figure 1:**
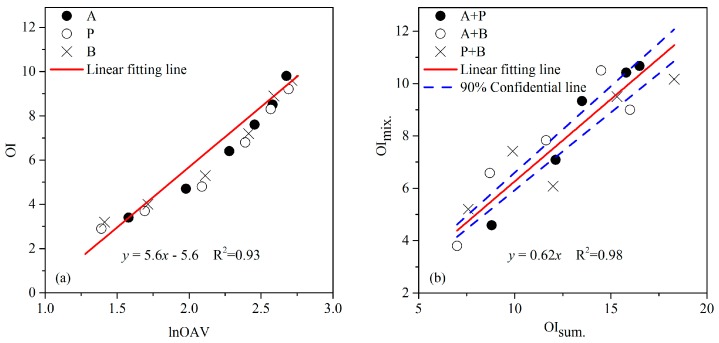
The (**a**) linear relation between odor intensity (OI) and logarithm of odor activity value (lnOAV) of individual aldehydes (acetaldehyde, A; propionaldehyde, P; *n*-butyraldehyde, B), and (**b**) the relationship between OI of a binary odor mixture (OI_mix._) and the summation of its unmixed constituents’ odor intensities (OI_sum._).

As illustrated in Figure 1a, the OI of individual aldehydes linearly increased with the increase of its lnOAV values. Furthermore, substances A, P, and B all matched well with the same formula (OI = 5.6lnOAV − 5.6). It was consistent with our previous research in aromatic hydrocarbons [25]. Actually, similar phenomenon also have been reported in literatures. As reported by Kim, a distinct linear relation between odor intensity and log(D/T) (i.e., logarithm of dilution-to-threshold (D/T) ratio which basically express a same meaning with the OAV) had been observed among individual sulfur compounds [34]. This was considered probably to be the consequence of their consistency in molecular structure and odor type. Because odor thresholds had been measured (Table 1) and it also was generally reported in literatures, OAV of stimulus could be easily calculated on the basis of its measured chemical concentration. Thus, odor intensity of individual acetaldehyde, propionaldehyde, and *n*-butyraldehyde could be directly predicted by employing the above formula. Furthermore, the same formula probably also suitable for other aldehydes with the same functional group and similar molecular structure.

Three binary odor mixtures of aldehydes (i.e., mixture of A and P, A and B, and P and B) were individually prepared as five different odor samples with odor intensity evenly distributed among level 1 to level 12 of the twelve-point OIRS. As shown in Figure 1b, the OI of a binary mixture (OI_mix._) apparently had a linear relation with the summation of its unmixed constituents’ odor intensities (OI_sum._). Similarly, these binary mixtures consisted of aldehydes all fitted the same formula (OI_mix._ = 0.62OI_sum._) well. According to the standard test method, interaction coefficient cosα (Equation (4)) in the vector model should be measured when two constituents in equal odor intensity levels (OI_a_ = OI_b_) were mixed. Thus, cosα of any binary mixture of acetaldehyde, propionaldehyde, and *n*-butyraldehyde was calculated by plugging the above formula in Equation (4):

(5) $$m=\cos\alpha_{\mathrm{ab}}=\frac{{\left(0.62\times \left({OΙ}_{a}+{OΙ}_{b}\right)\right)}^{2}-{OΙ}_{a}^{2}-{OΙ}_{b}^{2}}{2{OΙ}_{a}\cdot {OΙ}_{b}}=\frac{1.54{OΙ}_{a}^{2}-2{OΙ}_{a}^{2}}{2{OΙ}_{a}^{2}}=-0.22$$

Besides, the odor intensity of each constituent in Equations (1) and (2) could be displaced with its corresponding OAV by employing corresponding Equation (3) of aldehydes (i.e., OI = 5.6lnOAV − 5.6). Thus, by using the modified vector models, OI of any binary mixture or ternary mixture consisted of acetaldehyde, propionaldehyde, and *n*-butyraldehyde could be directly calculated on the basis of its constituents’ chemical concentrations. Therefore, it would be integrated with the optimal BP network for odor mixture’s OI predicting.

### 3.2. The MVM of Esters

**Figure 2:**
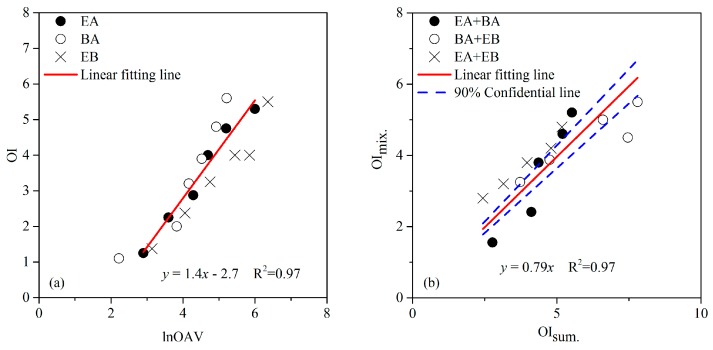
The (**a**) linear relation between odor intensity (OI) and the logarithm of the odor activity value (lnOAV) of individual esters (ethyl acetate, EA; butyl acetate, BA; ethyl butyrate, EB), and (**b**) the relationship between OI of a binary odor mixture (OI_mix._) and the summation of its unmixed constituents’ odor intensities (OI_sum._).

The results of ester’s MVM research were illustrated in Figure 2. Interestingly, similar linear relations like aromatic hydrocarbons and aldehydes were observed in both single esters (i.e., ethyl acetate, butyl acetate, and ethyl butyrate) and their binary mixtures. The linear fitting formula for odor samples of single EA, BA, and EB was OI = 1.4lnOAV − 2.7, and the formula for their binary mixtures was OI_mix._ = 0.79OI_sum._. Then, cosα of any binary mixture of ethyl acetate, butyl acetate, and ethyl butyrate was also calculated like in the following equation:

(6) $$m=\cos\alpha_{\mathrm{ab}}=\frac{{\left(0.79\times \left({OΙ}_{a}+{OΙ}_{b}\right)\right)}^{2}-{OΙ}_{a}^{2}-{OΙ}_{b}^{2}}{2{OΙ}_{a}\cdot {OΙ}_{b}}=\frac{1.54{OΙ}_{a}^{2}-2{OΙ}_{a}^{2}}{2{OΙ}_{a}^{2}}=-0.25$$

Based on the above results, the MVM of esters was also established for OI prediction.

**Figure 3:**
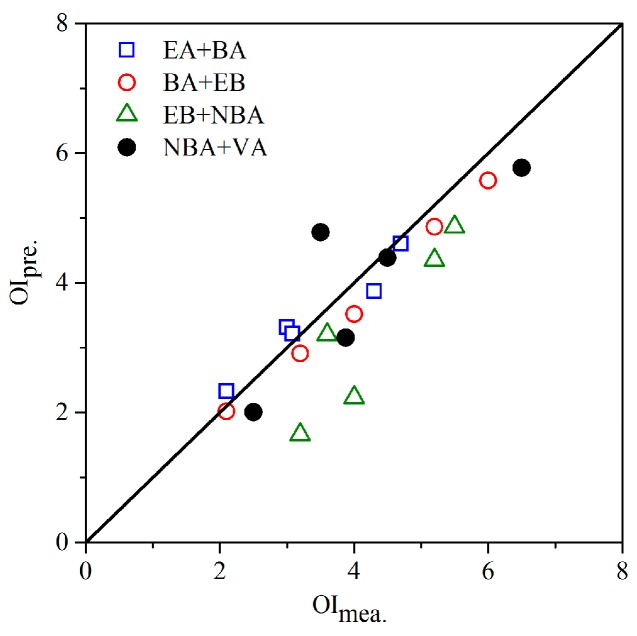
The comparison between measured OI (OI_mea._) and predicted OI (OI_pre._) of binary ester mixtures (ethyl acetate, EA; butyl acetate, BA; ethyl butyrate, EB; n-butyl acrylate, NBA; vinyl acetate, VA). The solid line means a complete match.

As depicted in Figure 3, a group of new odor samples of four different binary ester mixtures were evaluated by human assessors and their OI results were compared with the corresponding predicted ones. For mixtures consisted of EA, BA, and EB, the MVM showed a predictive accuracy well. It demonstrated the regular odor interaction pattern among odorants of the same type again. However, its predictive performance distinctly decreased when odor samples containing substance NBA or VA were tested. Actually, odorants NBA and VA had similar molecular structure with the other esters in this study. Besides, they also had a comparatively close odor type. Thus, this change was mainly attributed to their only difference of a hydroxyl group. It was in agreement with research results in the fields of chemoreception and chemosensory. As reported in the literatures, an odorant is recognized by a group of olfactory receptors and one olfactory receptor recognizes multiple odorants [35]. The relevance between specific receptors and a certain functional group had been widely observed and demonstrated [36,37]. For odorants with similar structure features (e.g., functional group), they probably shared more receptors. Thus, the same type of odorants exhibit more similarity in sensory stimulation, no matter if the single substance or their mixtures were tested. As a conclusion of the MVM research in aromatic hydrocarbons, aldehydes, and esters, factors including molecular structure, functional group, and odor type were proven to be key elements determining the odor interaction pattern in odor mixtures. As for their importance of odor interaction attributions, more specific investigations should be further performed.

### 3.3. Sensor Array and BP Network Optimization

**Figure 4:**
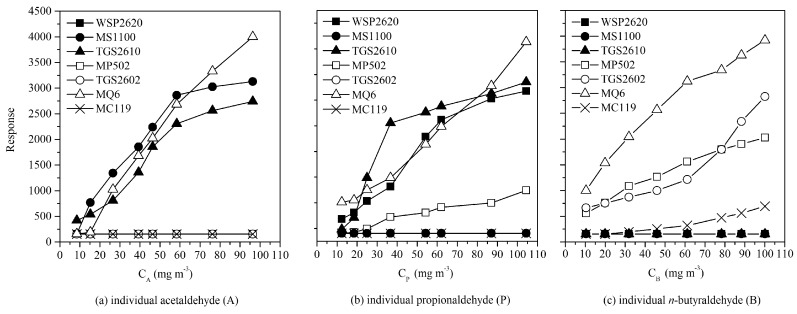
The concentration dependence of sensor response when single aldehydes were measured.

As shown in Figure 4, concentration dependence of sensors responses were plotted when a single aldehyde was measured. Gas sensors including TGS2602, TGS2610 (Tianjin Figaro Electronics Co., Ltd., Tianjin, China), MQ6, MC119, MP502, MS1100, and WSP2620 (Zhengzhou Winsen Electronics Technology Co., Ltd., Zhengzhou, China) were selected after testing because of their good linear response to one or some of the target substances. Among them, MQ6, MS1100, and TGS2610 expressed different levels of sensitivities to substance A while the others maintained at the background response level; WSP2620, MQ6, MP502, and TGS2610 expressed different sensitivities to substance P; MQ6, TGS2602, MP502, and MC119 expressed different sensitivities to substance B. Although, a specific sensor probably would simultaneously respond to several different stimuli, its sensitivities to them were still kept different. For instance, sensor MP502 apparently stronger responded to B in comparison with P. Thus, composition of an odor sample could be easily recognized from the sensor response pattern and the sensor array could provide sufficient information for further quantitative analysis.

After the above tests, a sensor array consisted of sensor TGS2602, TGS2610, MQ6, MC119, MP502, MS1100, and WSP2620 was finally established. Then, a new set of odor samples (*n* = 8 for A, P, B; *n* = 25 for each binary mixture; *n* = 50 for their ternary mixture) were measured by both the gas chromatography and the e-nose. The obtained chemical concentration and sensor signals of each sample were collected as a training database. In the following training procedure, sensor signals were used as target input of the BP network and chemical concentration of the corresponding sample was employed as its target output. In order to facilitate the convergence procedure of initial BP network, all training data were normalized before training [38]. In this study, sensor signals (target input) and chemical concentrations (target output) in the training database were separately normalized by using the following formula:

(7) $$y=\frac{x-\mathrm{MIN}}{\mathrm{MAX}-\mathrm{MIN}}$$

where *x* is the original data, y is the normalized data, MIN (MAX) is the minimum (maximum) of all the sensor signals or chemical concentrations. When employing the optimized BP network for sample analysis in further tests, all the sensor signals also should be normalized before processing and the corresponding output would be renormalized to obtain the real concentration value.

Except for the determined amount of neurons in both input layer and output layer, ‘logsig’ was selected as the transfer function between hidden layers, and ‘purelin’ was selected as the transfer function between the last hidden layer and output layer. After that, influences of training function, neuron quantity in hidden layer, and number of hidden layers were mainly explored in turn. The comparison results were shown in Table 3, average relative error (ARE) between the trained BP network’s output results and ideal ones was calculated for individual stimuli, binary mixtures, and ternary mixtures, respectively. Since the ARE was used as a descriptor of BP network’s predicting accuracy, parameters including training function ‘trainlm’, twenty neurons in each hidden layer and five hidden layers were finally determined on the basis of their corresponding minimum mean value of ARE (Table 3). Finally after training of a BP network with all the selected parameters, an optimized one was obtained and it would be employed for further tests.

**Figure 5:**
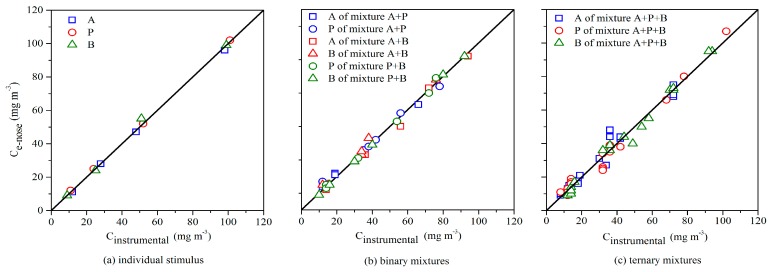
Comparison of measured chemical concentrations between the e-nose (C_e-nose_) and the gas chromatography (C_instrumental_). Odor samples consisted of acetaldehyde (A), propionaldehyde (P), and *n*-butyraldehyde (B).

In order to verify the analysis performance of the optimized BP network, comparison between gas chromatography analysis (C_instrumental_) and e-nose (C_e-nose_) was performed for a group of test samples (*n* = 4 for A, P, B; *n* = 6 for each binary mixture; *n* = 20 for their ternary mixture). From the results of e-nose testing, shown in Figure 5, one could see that the e-nose matched well with the traditional instrumental analysis (Pearson correlation coefficient was 0.999 for individual aldehydes, 0.996 for their binary mixtures, and 0.990 for their ternary mixtures). Hence, it laid a good foundation for odor intensity prediction.

### 3.4. Odor Intensity Assessment

**Figure 6:**
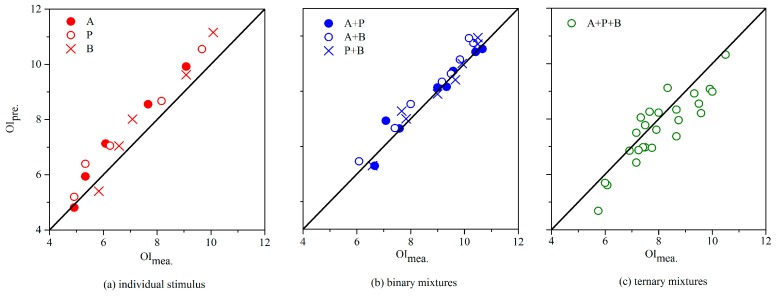
The comparison between measured OI (OI_mea._) and predicted OI (OI_pre._) of individual aldehydes (acetaldehyde, A; propionaldehyde, P; *n*-butyraldehyde, B), and their binary mixtures, ternary mixture. The solid line means completely match.

Through combination of the above selected sensor array, optimized BP algorithm and proposed aldehydes MVM, a new e-nose system was established for OI rating of aldehydes. After preparing a new group of odor samples (*n* = 5 for A, P, B; *n* = 8 for each binary mixture; *n* = 25 for their ternary mixture), odor intensity (OI_mea._) of each odor sample was firstly rated by the sensory panel in this study. Then, each odor sample was measured by the e-nose. Based on the obtained signal information from the sensor array, these signals were transformed into data and it was further processed by the optimized BP algorithm. As a result, the specific ingredients of an odor sample and chemical concentration of each constituent were all provided. These obtained results were substituted in the proposed aldehydes MVM, and OI of corresponding sample was calculated (OI_pre._). As shown in Figure 6, the e-nose (OI_pre._) slightly overestimated for individual stimuli than human assessors (OI_mea._) with Pearson correlation coefficient of 0.980 which indicates the above two groups of results were positively linearly correlated. It matched well for binary mixtures (Pearson correlation coefficient of 0.973) and slightly underestimated for ternary mixture (Pearson correlation coefficient of 0.888). Taking into consideration of the instability in human assessors, the scatter of evaluation results in a limited range (e.g., 0.5 of the OIRS) is generally acknowledged. Thus, the overall e-nose performance was considered adequate for the application in odor intensity assessment.

Comparing with traditional instrumental analysis and olfactory evaluation, the e-nose might perform less well in precision by now. However, e-nose takes advantage of quick response and portable application, and it just meets the requirements of odor pollution assessment and odor source tracing in actual applications. Besides, the participation of human assessors had been avoided as far as possible by employing the combination of ANN and empirical functions. Thus, it would distinctly save both costs and time consumption in odor intensity rating. On the other hand, research found that the OI of a complex mixture was mainly contributed to only several ingredients and the other ingredients barely influenced it [16,39]. Thus, OI predicting of complex mixtures not always requires the same complex empirical function. Therefore, developing the combination method between an ANN algorithm and empirical functions is a promising way to facilitate the extensive application of e-nose.

## 4. Conclusions

This study investigated the feasibility of a modified vector model (MVM) in two different groups of odor mixtures which consisted of aldehydes and esters, respectively. Through a series of validation tests, the regular odor interaction pattern among odorants of the same type was demonstrated and it was mainly attributed to their common features including molecular structure, functional group, and odor type. The MVM successfully transformed quantitative analysis results of an odor sample into its corresponding odor intensity. Compared with other empirical functions in the literature, it kept predictive accuracy well and even expanded its application scope. The proposed new e-nose system successfully combined the MVM with an optimized BP algorithm, it fulfilled the demands of quantitative analysis and OI rating at a same time. After a comparative study of the e-nose system and traditional methods (i.e., gas chromatography and human assessor), the e-nose performed well in both quantitative analysis and odor intensity assessment. As a conclusion, the regular interaction pattern provided a foundation for modification of empirical models and it further accelerated the development of e-nose and its applications in air quality monitoring.

## Figures and Tables

**Table 1:** List of odorants used for odor sample preparation.

Order	Odorant (Abbreviation)	CAS#	Chemical Structure	Reported Odor Threshold/ (mg/m^3^)	Measured Odor Threshold ^v^/ (mg/m^3^)
1	Acetaldehyde (A)	75-07-0	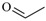	0.003 ^I^/0.366 ^II^	0.039
2	Propionaldehyde (P)	123-38-6	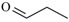	0.002 ^I^/0.376 ^II^	0.041
3	*n*-Butyraldehyde (B)	123-72-8	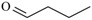	0.002 ^I^/0.029 ^II^	0.052
4	Ethyl acetate (EA)	141-78-6	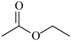	3.422 ^I^/2.399 ^II^	0.276
5	Butyl acetate (BA)	123-86-4	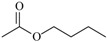	0.083 ^I^/0.034 ^II^	0.085
6	Ethyl butyrate (EB)	105-54-4	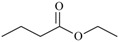	0.2 × 10^−3 I^/0.005 ^III^	0.053
7	*n*-Butyl acrylate (NBA)	141-32-2	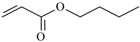	0.017 ^II^/0.003 ^IV^	0.038
8	Vinyl acetate (VA)	108-05-4	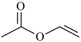	2.318 ^II^/0.462 ^IV^	0.072

^I^ Odor thresholds in reference [28]; ^II^ Odor thresholds in reference [29]; ^III^ Odor thresholds in reference [30]; ^IV^ Odor thresholds in reference [31]; ^v^ Odor thresholds measured by the sensory panel in this study with a standard method [32].

**Table 2:** Odor intensity referencing scales (OIRS).

	Odor Intensity Levels						
	1	2	3	…	8	…	12
12-Point Scale	<10>	<20>	<40>	…	<1280>	…	<20480>
8-Point Scale	<12>	<24>	<48>	…	<1550>	-	-

<PPM>: water solution of *n*-butanol (ppm) with a geometric concentration progression of two. Temperature of water solution maintains at 27 ± 1 °C.

**Table 3:** The average relative error of BP neural network with different parameters.

Subjects	Average Relative Error (ARE, %)			
	Individuals (*n* = 24)	Binary Mixtures (*n* = 75)	Ternary Mixtures (*n* = 51)	Mean Value
a. Training functions				
trainlm	5.0	10.0	19.4	11.5
traingd	6.0	10.1	21.0	12.4
traingdm	5.4	11.2	20.5	12.4
traingdx	6.0	10.2	26.2	14.1
traingda	8.0	12.1	21.7	13.9
trainrp	8.9	12.5	19.6	13.7
b. Neuron quantity in the hidden layer				
12	5.0	10.0	19.4	11.5
14	4.0	8.2	25.8	12.7
16	4.0	13.4	22.5	13.3
18	13.5	15.0	28.6	19.0
20	4.0	8.5	20.1	10.9
22	4.2	8.9	24.5	12.5
c. Quantity of hidden layers				
3	5.0	10.0	19.4	11.5
4	4.5	8.0	17.8	10.1
5	3.2	8.1	11.9	7.7
6	4.6	7.3	13.9	8.6
7	5.2	8.8	17.6	10.5
